# Examining intrafamilial communication of colorectal cancer risk status to family members and kin responses to colonoscopy: a qualitative study

**DOI:** 10.1186/s13053-019-0114-8

**Published:** 2019-06-26

**Authors:** Kaitlin M. McGarragle, Crystal Hare, Spring Holter, Dorian Anglin Facey, Kelly McShane, Steven Gallinger, Tae L. Hart

**Affiliations:** 1 Ryerson University, 350 Victoria Street, Toronto, ON M5B 2K3 Canada; 2 Zane Cohen Centre for Digestive Diseases, Sinai Health System, Box 24-60 Murray Street, Toronto, ON M5T 3L9 Canada; 3 Department of General Surgery, Toronto General Hospital, 200 Elizabeth St., 10EN, Room 206, Toronto, ON M5G 2C4 Canada; 4 Department of Psychiatry, University of Toronto, 250 College Street, Toronto, ON M5T 1R8 Canada

**Keywords:** Colorectal cancer, Disclosure, First-degree relatives, Colonoscopy adherence, Screening, Stages of change

## Abstract

**Background:**

First-degree relatives (FDRs) of probands with colorectal cancer (CRC) may be at increased risk of CRC and require colonoscopy. Proband disclosure about this risk and need for colonoscopy is essential for FDRs to take appropriate action. Low colonoscopy rates are reported among FDRs and little is known about the proband disclosure process. A better understanding of the barriers surrounding colonoscopy and disclosure is needed.

**Methods:**

CRC probands (*n* = 16) and FDRs (*n* = 9), recruited from a Canadian CRC Consortium, completed interviews to determine barriers to disclosure and colonoscopy, respectively. Interviews were analyzed using thematic analysis and participants’ motivation to disclose to FDRs or undertake colonoscopy was categorized into Stages of Change (i.e., Precontemplation, Contemplation, Preparation, Action, or Maintenance) using the transtheoretical model.

**Results:**

25% of probands had not disclosed to any first-degree kin and were categorized in the Precontemplation or Contemplation Stage of Change. Barriers to disclosure included lack of information, negative expectations about familial reaction, assuming FDRs were aware of risk or already being screened, dysfunctional family dynamics, and cultural barriers. 75% of FDRs were categorized in the Precontemplation or Contemplation Stage of Change. Barriers included negative perceptions about colonoscopy, health-care provider related factors, practical concerns, and lack of information about CRC, risk, and colonoscopy.

**Conclusions:**

In the absence of barriers such as cost and accessibility, this Canadian sample still reported several challenges to disclosure and colonoscopy adherence. Future research should explore interventions such as motivational interviewing to improve proband disclosure and to increase FDR adherence to colonoscopy.

## Background

Colorectal cancer (CRC) is the second most common cancer in Canada and the fourth most common in the United States [1, 2]. In 2014, nine Canadian provinces implemented CRC screening programs to facilitate early detection and encourage regular screening for average risk individuals. However, programs have struggled to meet the screening target rate of 60% [2], with some studies documenting uptake rates of colonoscopy ranging from 22 to 40% among first degree relatives (FDRs) with a family history of CRC [3–5].

A recent review of qualitative studies cited the following barriers to FDRs’ compliance with colonoscopy recommendations: negative attitudes towards screening (e.g., pain, discomfort, embarrassment), fear of abnormal test results or diagnosis of cancer, procedure cost, limited accessibility to healthcare resources, lack of awareness of increased CRC risk, external locus of control resulting in a lack of interest in colonoscopy, and time constraints associated with the preparatory and screening process [6]. Studies have also highlighted predictors of regular colonoscopy in FDRs such as younger age [7], recommendation from a health-care provider (HCP) and a close, supportive relationship between FDRs and their relative(s) diagnosed with cancer [3, 8–10].

The burden of informing FDRs about their CRC risk and need for colonoscopy often falls upon the individuals diagnosed with CRC [11]. Although limited research exists examining this discourse for people with CRC and their kin, recent studies have documented obstacles to disclosure among people diagnosed with Lynch syndrome (LS). Barriers to informing relatives in this population include the assumption that another family member may have told them about their increased risk, difficulty discussing the topic of cancer, feeling the responsibility is too emotionally demanding, not feeling the information is relevant for a particular relative (e.g., based on young/old age), fear of harming the relationship, being socially distant with the relative and/or having negative relationships with certain relatives [12–15]. Regarding communication between individuals with CRC and their FDRs specifically, one study from Singapore found that individuals with CRC tend to misunderstand colonoscopy guidelines for FDRs, believe colonoscopy is a forbidden subject, and report not receiving advice from HCPs on how to talk to their relatives about colonoscopy [11]. To improve colonoscopy adherence, it is imperative to understand kin-reported barriers to colonoscopy and issues in intrafamilial communication.

A useful model for understanding barriers to modifying health behaviors is the transtheoretical model (TTM), which has been successfully used to increase physical activity, smoking cessation, mammography screening, and more recently, CRC screening [16–19]. The TTM provides categories for understanding an individual’s “Stage of Change,” or readiness to take action [20]. In the Precontemplation stage, individuals have no intention of changing their behavior in the future (usually the next 6 months). In the Contemplation stage, individuals intend to change their behavior in the next 6 months however, this stage involves considerable ambivalence. In the Preparation stage, individuals intend to change their behavior immediately, usually in the next month and already have a strategy. In the Action stage, individuals have already changed their behavior, usually in the last 6 months. In the Maintenance stage, individuals are confident in their ability to continue engaging in the behavior.

The Stages of Change have been empirically linked to colonoscopy uptake. For example, Wang et al. found that 90% of individuals who had never had a colonoscopy or who had one in the distant past were in the Precontemplation stage [21]. These individuals reported experiencing unique barriers to colonoscopy adherence (i.e., lower perceived benefits, lower self-efficacy) compared to individuals in more advanced stages (e.g., Action, Maintenance). In a similar study, individuals with a family history of CRC in the Precontemplation stage reported significantly more barriers than those in other stages [22]. These findings indicate the existence of underserved and unmotivated populations that are at increased risk of developing CRC but fail to benefit from the possible life-saving effects of routine colonoscopy. Importantly, researchers have noted that their barriers are not targeted by generic interventions (e.g., postcard screening reminders) [16].

While prior research has elucidated FDRs lack of motivation to obtain colonoscopy for reasons such as lack of a physician recommendation and lack of insurance [8], this study examined a unique group of participants who all had access to provincial healthcare. Moreover, all individuals with CRC in the current study had been diagnosed under the age of 60, suggesting a more immediate need to inform family members earlier. Individuals with CRC are often at the front line of communicating critical information to family members, but it is clear significant barriers can preclude this from happening. The aim of this study was to utilize the TTM to clarify the barriers that exist at each stage of change for (1) Individuals with CRC struggling with disclosing to their FDRs that they may be at increased risk of developing CRC and need regular colonoscopy, and for (2) FDRs who fail to get regular colonoscopy.

## Methods

### Participants

Participants were recruited from the Canadian Colorectal Cancer Consortium (C4); a multi-site CRC research study. The C4 prospectively recruited individuals diagnosed with incident CRC under age 60 and their at-risk FDRs from six sites across Canada (Mount Sinai Hospital, Toronto, ON; Sunnybrook Health Sciences Centre, Toronto, ON; St. Michael’s Hospital, Toronto, ON; McGill University Health Centre, Montreal, QC; Alberta Health Services, Edmonton, AB; St. Paul’s Hospital, Vancouver, BC). Research Ethics Board approval was obtained at each site. Consented probands provided access to their CRC treatment records, had LS tumor screening and subsequent germline genetic testing if necessary, and completed family and personal history questionnaires. Families were risk-classified as high-germline risk (LS or Familial Colorectal Cancer Type X), intermediate-germline risk (CRC diagnosed < 35 years, CRC < 50 with FDR CRC < 50, or multiple primary CRCs), or low-germline risk (proband-only CRC diagnosis at older age). Probands were also asked to complete a Family Address Sheet (FAS) to obtain contact information for their eligible FDRs who were mailed an invitation letter describing the C4, as well as a reply form which they could mail back to express interest in participating. Interested FDRs spoke to a study coordinator and if agreeable to study procedures, were mailed an informed consent and personal history questionnaire which included questions regarding colonoscopy. All consented FDRs were provided with written colonoscopy recommendations based on their family risk classification and age. A letter with colonoscopy recommendations was also provided to FDRs’ family physicians, if available, at half the C4 sites. All data were recorded in the C4 database. Consented participants were asked to allow future contact from the C4 for additional Research Ethics Board approved studies.

### Procedure

C4 probands were approached for participation for this study when they did not complete the FAS, which would have allowed the study team to contact their FDRs about participating in the C4. This was used as a proxy to select participants who were unlikely to have told their relatives about their CRC risk. FDRs who had consented to participate in the C4 and who had not undergone colonoscopy in the past 10 years were approached. All participants provided written informed consent and were offered a choice between an interview conducted via telephone or focus group. Individual interviews lasted 30 min on average. One focus group was convened, with four participants, and lasted 60 min. The focus group was facilitated by a genetic counselor and two clinical psychologists who had expertise in working with this population and were trained in qualitative data collection techniques. All interviews were audio-recorded and transcribed.

### Assessment

A series of qualitative research questions were developed based on Paddison and Yip’s exploratory study that investigated the barriers to CRC screening [23]. These questions have been tailored to each Stage of Change in the model developed by Prochaska et al. [19]. For FDRs, questions assessed beliefs, benefits, risks, barriers, and facilitators to colonoscopy. For CRC patients, questions assessed beliefs, barriers, and facilitators to intrafamilial disclosure about CRC-related information, particularly with regard to colonoscopy.

### Data analysis

Interview coding was performed by two trained master’s level students, who were supervised by two senior researchers, one with expertise in CRC and one with expertise in qualitative data analysis. All members of the research team had prior training in qualitative data analysis. The research team met weekly to review the coding process until saturation was reached. Thematic analysis was used to analyze interview transcripts [24]. Two authors (KMM, CH) reviewed all transcripts separately using an inductive approach. Transcripts were first read several times and preliminary notes were recorded. The TTM was used to categorize participants into their appropriate Stage of Change. Quotes relevant to the present study’s research objectives were highlighted. Similar quotes were then grouped together into common themes and sub-themes and an initial codebook was developed. The study team reviewed the initial codebook and made revisions based on overlapping codes to obtain parsimony. When disagreement occurred between the two authors regarding specific themes or codes, the two senior researchers were consulted (TLH, KM). The final codebook was reviewed and agreed upon by members of the research team. Interviews were conducted until saturation was reached, which occurred at interview 10 for probands and interview 8 for FDRs. Additional interviews were undertaken as they were previously scheduled prior to establishing saturation.

## Results

### Participant characteristics

**Fig. 1:**
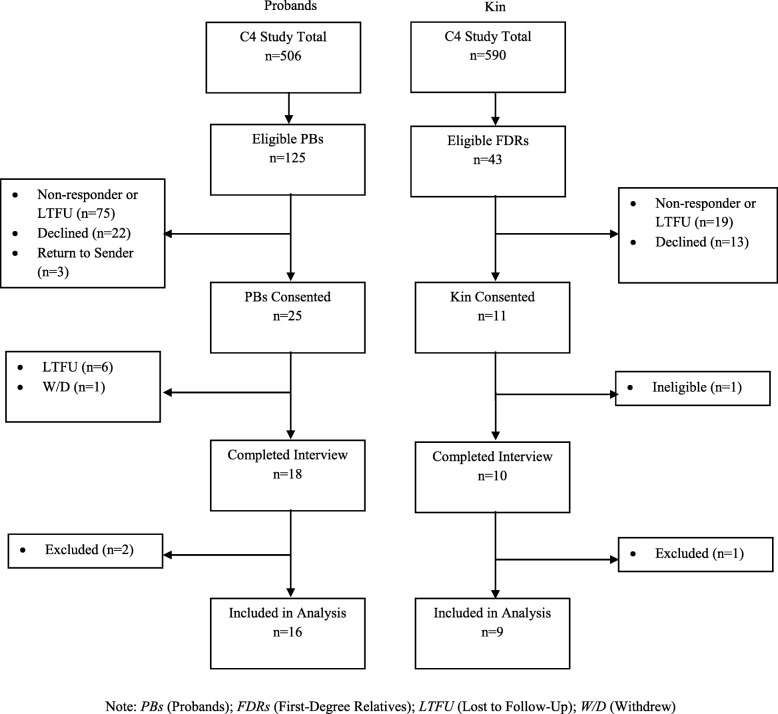
Participant Recruitment Summary. Note: *PBs* (Probands); *FDRs* (First-Degree Relatives); *LTFU* (Lost to Follow-Up); *W/D* (Withdrew)

Participant recruitment details are displayed in Fig. 1. Proband response rate was 14.4%, and FDR response rate was 23.2%. A total of 18 probands and 10 FDRs were interviewed. Two probands and one FDR were excluded from analysis for not meeting the eligibility criteria. Both probands disclosed during interviews that they were adopted and therefore did not know their biological family’s cancer risk status, and the kin participant was younger than the recommended age to begin colonoscopy screening based on her family history of CRC. Sixteen probands and nine kin interviews were included in analysis. Participant demographics and clinical characteristics are displayed in Table 1. The mean age of probands (*n* = 16) was 52, and the mean age of FDRs (*n* = 9) was 56. Our sample was mostly white, female, married, and employed with at least a college/university education or higher. A minority of participants had LS, and most FDRs were at low germline risk for CRC.

**Table 1:** Participant Demographic and Clinical Characteristics

	Probands (*n* = 16)	Kin (*n* = 9)
	% (N)	% (N)
Current Age (Mean, SD)	51.9 (9.56)	56.00 (14.14)
Age at Diagnosis (Mean, SD)	48.9 (9.06)	–
Gender		
Male	43.8 (7)	44.4 (4)
Female	56.3 (9)	55.6 (5)
Marital Status		
Married	56.3 (9)	88.9 (8)
Education		
High School or Voc/Tech	18.8 (3)	44.4 (4)
Some College/University	43.8 (7)	11.1 (1)
Bachelor’s Degree	31.3 (5)	22.2 (2)
Graduate Degree	0 (0)	22.2 (2)
Employment Status		
Unemployed	12.5 (2)	11.1 (1)
Employed	75.0 (12)	66.7 (6)
Retired	12.5 (2)	22.2 (2)
Ethnicity		
White	68.75 (11)	100 (9)
Asian	18.75 (3)	0 (0)
Hispanic	6.25 (1)	0 (0)
Multi	6.25 (1)	0 (0)
Lynch Syndrome		
Yes	18.75 (3)	0 (0)
Germline Risk Classification		
Low	56.3 (9)	77.8 (7)
Intermediate	25.00 (4)	22.2 (2)
High	18.8 (3)	0 (0)
Stage		
1	6.3 (1)	–
2	56.3 (9)	–
3	31.3 (5)	–
4	6.3 (1)	–
Chemotherapy		
Yes	56.8 (9)	–
Number of FDRs	4.94 (2.79)	–
Letter sent to GP?		
Yes	–	55.6 (5)

Note: *SD* Standard Deviation, *N* Total Number of Participants, *Voc* Vocational, *Tech* Technical, *FDRs* First-Degree-Relatives, *GP* General Practitioner

### Proband disclosure: stages of change

Proband Stages of Change are displayed in Table 2. Most probands fell into one Stage of Change however, some fell into two stages when they had disclosed to some FDRs but not others. Stage(s) of Change is provided with participant quotes.

**Table 2:** Stages of Change, Probands (*N* = 16)

Pt ID, Gender	Current Age	Informed of Increased Risk?	Informed FDRs?	Stage of Change
PB#13, Female	48	Yes	No	PRECONTEMPLATION − Has not informed only living FDR (mother), does not intend to − Anticipates negative reaction
PB#5, Male	62	Yes	No	PRECONTEMPLATION − Estranged from family, does not intend to disclose to any FDRs
PB#7, Male	62	No	No	PRECONTEMPLATION − Would not consider discussing increased risk with brother however, brother is aware of cancer diagnosis − Believes brother already undergoing routine colonoscopy due to age (> 50) CONTEMPLATION − Would consider disclosing to children after being informed of risk BUT perceives their younger age as barrier
PB#4, Female	48	Yes	One	ACTION − Informed mother only about increased risk PRECONTEMPLATION − Does not plan to disclose to any other FDRs or revisit conversation with mother − Cultural and informational barriers to family communication
PB#6, Male	56	Yes	Most	MAINTENANCE − Has disclosed to most siblings, children and willing to revisit conversation PRECONTEMPLATION − Has not disclosed to brother living outside of Canada, does not intend to (believes other family members have informed him)
PB#24, Male	61	No	No	CONTEMPLATION − Not advised by HCPs to disclose to FDRs − Would consider telling them after learning this information
PB#3, Male	54	Yes	Some	MAINTENANCE − Has told male relatives and continues to remind them to get screened PREPARATION − Was not aware females underwent colonoscopy − Intends to inform female relatives after learning they are eligible for screening
PB#10, Female	36	Yes	Yes	ACTION − FDRs already aware of risk due to family history but has informed them since diagnosis
RF#1, Female Lynch	56	Yes	Yes	ACTION − “One-time conversation” with family members
PB#15, Female	46	Yes	Yes	ACTION − No children or siblings BUT has informed cousins, parents − Has not revisited conversation (family members avoid talking about it)
PB#17, Female	63	Unsure	Yes	ACTION − Has told siblings, mother (living relatives)
PB#23, Male	57	Yes	Yes	MAINTENANCE − Has informed siblings, parents − “Kept on them” about colonoscopy post-disclosure
RF#2, Female Lynch	39	Yes	Yes	MAINTENANCE − Has told all FDRs − Continues to follow-up about screening
RM#1, Male	59	Yes	Yes	MAINTENANCE − Has followed up with male family members BUT uncomfortable following up with female relatives
PB#16, Female	33	Yes	Yes	MAINTENANCE − Has told only sibling (brother), parents − Followed up with brother post-disclosure − Children too young to be screened
PB#22, Female	50	Yes	Yes	MAINTENANCE − Told and followed-up with siblings, parents − Children too young to be screened

Note: *Pt* Participant, *PB* Proband, *HCPs* Healthcare Providers, *FDRs* First-Degree-Relatives

Twenty-five percent of probands (*n* = 4) had not disclosed to any FDRs and were categorized as being in Precontemplation or Contemplation. Of these probands, only half were informed by HCPs about the need to advise FDRs about the increased risk of CRC and the need to undergo colonoscopy.

Approximately 31% of probands (*n* = 5) were categorized as being in the Action stage. Most probands in Action (*n* = 4) reported disclosing to all FDRs during a one-time conversation and the remaining one proband had told only one FDR. Probands in the Action stage did not report revisiting the conversation about CRC screening with their FDRs or following up with them regarding colonoscopy.

Almost half of probands (*n* = 7) were categorized as being in the Maintenance stage and reported multiple conversations with FDRs in which they followed up about colonoscopy. Of the seven probands in Maintenance, five had disclosed to all their FDRs. One of the seven reported also being in the Preparation stage with his female relatives, as he was unaware of the need for women to undergo colonoscopy prior to being interviewed. This same proband had already informed all of his male relatives. The remaining proband was in Precontemplation about disclosing to one FDR with whom he was no longer in contact with that lived outside of Canada.

### Probands barriers to disclosure

#### Lack of information

Many probands discussed lack of information as a barrier to disclosure. Some probands reported not being informed by HCPs about the need to advise FDRs about their increased CRC risk and need for colonoscopy. Many others discussed a lack of information regarding CRC screening guidelines in general, and on how to talk to family members about CRC risk and colonoscopy. As noted above, one proband discussed being unaware that female FDRs should be advised to undergo colonoscopy.

*I wasn’t sure if the ladies as well as men get colonoscopy.* (PB #3, Preparation and Maintenance)

#### Negative expectations about familial reaction

Many probands in Precontemplation discussed having negative expectations about the way their FDRs would react to being told to get a colonoscopy. Probands talked about discomfort surrounding the topic of CRC and colonoscopy that made disclosure challenging and reported anticipating embarrassment on behalf of FDRs.

In addition, when probands perceived a lack of baseline knowledge about cancer that made discussing CRC screening and increased risk challenging, it acted as a barrier to disclosure. For example, one proband described disclosing to only one FDR and not others, assuming other FDRs would negatively react in a similar way.

*...there’s one thing that she said, ‘Oh, is that cancer contagious?’ It’s like, I said, oh my god, I flipped, I really flipped when I heard that... I said, no, this is not like a flu or something. So at that moment I said, that’s it, I’m not going to deal with this, deal with this just with them explaining, because they don’t know anything.* (PB #4, Precontemplation and Action)

Probands further remarked on an assumption that FDRs would be unwilling to change and some talked about disclosure being pointless. Probands assumed that based on younger or older age (≥65), FDRs would not be receptive to this information. For example, one proband described reluctance in disclosing to her father:

*My dad… he’s just very set in his ways. Old school, I guess.* (PB #10, Action)

#### Assumption that FDRs are aware of risk or already being screened

Another common barrier to disclosure was the assumption that FDRs were already aware of their CRC risk or already undergoing routine CRC screening. For example, probands discussed believing that other family members had informed their FDRs:

*Yeah. I personally didn’t let him know, but I can guarantee you that there are a few family members that he does talk to... I know for sure my oldest brother would have told him.* (PB #6, Precontemplation and Maintenance)

Probands assumed because their family members attend regular visits with their general practitioner (GP) or because they are ≥50, they are already undergoing regular CRC screening. For example, one proband mentioned a sibling who he assumed was already undergoing regular screening because of his age:

*He gets his testing done. I don’t know that it’s something he wants to talk to me about... Especially in these days, you know, everybody, they do get tested.* (PB #7, Precontemplation and Contemplation)

#### Dysfunctional family dynamics

Probands commented on dysfunctional family dynamics, such as not being in touch with FDRs, lack of closeness, lack of openness, concerns about negative reactions, and estrangement as barriers to disclosure. Probands described reasons such as physical distance and gradual contact attenuation for not being in touch with FDRs.

An example of lack of closeness was provided by one proband who reported not being in touch with family members who lived far away:

*I’m not really that much in touch with them... I think they’re a little bit too far away. You know, you don’t want to discuss something when they don’t even live here...discussing cancer to them, it’s not that easy* (PB #7, Precontemplation and Contemplation)

Other probands stated they were worried about hostile reactions and chose to not disclose to FDRs because they did not want to deal with the fall-out. For example, one proband described why they decided not to disclose to their mother:

*I don’t want her to overreact and just become overly fussy with me, and she would try and take command of the issue and tell me what I should and shouldn’t do. And get on top of it and dictate, and to treat me as if I’m not aware of the situation and on top of it myself.* (PB #13, Precontemplation)

Many probands in Precontemplation also reported estrangement from FDRs who they had not disclosed to:

...*we are partially estranged so I don’t want to discuss my health issues with her.* (PB #13, Precontemplation)

*The other reason is, after being estranged all these years, I do not want them to think I’m trying to contact them for sympathy* (PB #5, Precontemplation)

#### Cultural barriers

Some probands reported cultural barriers to disclosure that made discussions about their own cancer challenging, as well as discussions about the need for cancer screening. For example, one proband who did disclose to her FDRs but found following up with them about colonoscopy adherence very difficult described how the topic of cancer was proscribed among her family members:

*…in my father’s side of the family, things like this are not discussed. The cancer word is not used…it is not something that would have been discussed with the kids anyway. Not in our culture at least.* (PB #15, Action)

Another proband mentioned that preventative health measures were not endorsed in her culture:

*No, they just, like, because back home it’s [only] when you’re sick you go [to seek health care]* (PB #4, Action and Precontemplation)

### FDR adherence to colonoscopy: stages of change

FDR Stages of Change are displayed in Table 3. Five of nine FDRs were categorized as being in the Precontemplation stage and were not considering undergoing colonoscopy. Two of the five FDRs in Precontemplation discussed undergoing regular fecal occult blood tests (FOBT) instead of colonoscopy. The remaining three FDRs reported that no HCP had recommended colonoscopy to them, and only one of the three had a GP who had received a letter about their increased risk for CRC. Lack of information and distress about the preparation associated with colonoscopy were primary concerns at this stage.

**Table 3:** Stages of Change, Kin (*N* = 9)

Pt ID, Gender	Relative w/ CRC	Current Age	Time Since PB dx	Ready to Take Action?	GP Letter?	Stage of Change	Beliefs about Colonoscopy	Key Barriers to Colonoscopy
Kin#8, Female	Brother	52	2.5 yrs	No^c^	Yes	PRE-CONT	− Not a pleasant procedure − Requires extensive preparation − Similar to pap smear − Preventative	− GP recommended stool test based on age (> 50) − Negative beliefs about colonoscopy − Spouse had bad experience − Misinformation (no need for colonoscopy b/c asymptomatic)
Kin#9, Male	Sister	44	3 yrs	No	No	PRE-CONT	− Not worried about procedure − Preventative BUT − Ignorance is bliss	− No GP − Lack of info/unsure where to find reputable info − Desire for more info on risk − No motivation (self-described laziness)
Kin#14, Female	Mother	42	4 yrs	No	Yes	PRE-CONT	− Preparation “often worse than procedure itself” − Preventative	− HCPs have not recommended colonoscopy (mother did) − Scheduling − Negative beliefs about colonoscopy − Misinformation (no need for colonoscopy b/c asymptomatic) − Privacy concerns
Kin#19, Female	Son	83	4 yrs	No	No	PRE-CONT	− Not convinced it is necessary − Not appealing (enema) − Risks do not outweigh benefits	− HCPs have not recommended colonoscopy − Perceived risk is low (“generation removed”) − Negative beliefs about colonoscopy
Kin#25, Female	Sister	54	4 yrs	No^c^	Yes	PRE-CONT	− Stool test first − Preparation unappealing: drink beforehand “biggest reason” for not undergoing	− GP recommended stool test based on age (> 50) − BUT “if [GP] told me I had to do it, I would do it.” − Misinformation (stool test sufficient) − Mimicking screening behaviors of FDRs − Desire for more info on risk − Negative beliefs about colonoscopy
Kin#2, Male	Father	41	4.5 yrs	Maybe	Yes	CONT	− Preventative − Colonoscopy “on the radar”, needs to do it	− Fear of CRC − Scheduling (previously in PREP stage) − Practical (i.e. bad weather) − GP not informed of risk/family history
Kin#18, Female	Brother	53	1.5 yrs	Maybe^a^	No	CONT	− Preventative BUT − Big time commitment (preparation and recovery) − High priority, keeps thinking about it	− Scheduling − Does not understand risk and desires more specific info
Kin#1, Male	Daughter	65	4 yrs	Yes	Yes	PREP	− Proactive and part of a healthy lifestyle − Offers peace of mind − Preparation unappealing: drink beforehand	− Negative beliefs about colonoscopy preparation − Perceived risk on spouse’s side
Kin#26, Male	Brother	70	4 yrs.	Yes^b^	No	ACTION	− Preventative measure: removes polyps, can improve bowel function − Previously “disgusted” by idea of colonoscopy	− N/A

^a^underwent colonoscopy prior to family member’s diagnosis

^b^underwent colonoscopy post-family member’s diagnosis

^c^has had stool test

Note: *Pt* Participant, *w/* with, *yrs* years, *CRC* Colorectal Cancer, *GP* General Practitioner, *PRE-CONT* Precontemplation, *CONT* Contemplation, *PREP* Preparation, *dx* diagnosis

Two of nine FDRs were categorized as being in the Contemplation Stage of Change. Neither FDR expressed definitive knowledge about their level of CRC risk; however, both were advised by a HCP to undergo colonoscopy based on their family history.

The remaining two FDRs were in the Preparation and Action stages, respectively. The FDR in Preparation agreed to discuss colonoscopy with his GP because of his age (≥50) but had not yet agreed to set a colonoscopy date. He discussed mostly neutral and positive beliefs about colonoscopy but did express concern about the preparation associated with it, particularly the drink patients are required to consume beforehand. The participant in the Action stage was included because at the time of recruitment, he had not yet undergone colonoscopy. However, once the interview was scheduled, he had completed it, yet was 4 years overdue. The primary reason he decided to proceed with colonoscopy was the sight of blood in his stool. He was included to undercover additional reasons for previously avoiding colonoscopy.

### Barriers to colonoscopy

#### Negative attitude about colonoscopy

Many FDRs revealed negative attitudes about the preparation associated with colonoscopy and/or the procedure itself. These attitudes were founded on information gathered from family members or friends who had undergone the procedure. For example, many FDRs discussed how unappealing they perceived the enema, sedation, and drink affiliated with colonoscopy to be.

*I thought about it but if they make me drink that stuff, I’ll just throw it up. I’ve tasted it and I just couldn’t drink it, it wouldn’t stay down. I just had a sip... I would say yah that would be the biggest reason*. (Kin #25, Precontemplation –GP sent letter)

*“...the idea of the whole procedure disgusted me…. just the thought of it made me not want to do it… even if that meant I might die. It just seemed so gross in my mind before the procedure. I had images of being in some really obscene posture while they stuck something into my rear end. And in a nutshell, that’s it. And to have to go through that, you know. I think you’d have to almost be at death’s door.”* (Kin #26, Action -GP not sent letter)

One FDR brought up fear of contamination regarding colonoscopy and talked about her spouse’s negative experience undergoing the procedure.

*When my husband did go in, I think it was a year or two later they sent him a letter saying that it had been discovered that the tools had not been cleaned properly, so if you had any issues … And I was like, oh, that’s nice… So, that definitely kind of sticks in my mind.* (Kin #8, Precontemplation –GP sent letter)

#### Lack of motivation

Some FDRs discussed an overall lack of motivation to undergo colonoscopy or attend general health check-ups. For example:

*I’ve been putting it off. How about that? I haven’t decided or undecided. I guess by putting it off, that means I’ve decided not to.* (Kin #8, Precontemplation -GP sent letter)

#### HCP-related factors

Many FDRs remarked on several HCP-related factors that acted as barriers to colonoscopy. Uninformed HCPs for example, could not recommend colonoscopy because they were unaware of the CRC family history. Similarly, FDRs who reported not having a GP could not have been referred for colonoscopy.

Incorrect information provided to some FDRs by their HCPs also acted as a barrier to colonoscopy. For example, two FDRs chose to undergo FOBT in lieu of colonoscopy. This decision was made because they were informed by their HCPs that if concerning findings were discovered during, they could choose then to proceed with colonoscopy.

*But he said no, we’ll do the test first. If they find anything, then we can go further with the colonoscopy. So, that was another reason I thought, well, then maybe I can get away with just that.* (Kin #8, Precontemplation –GP sent letter)

*But I just did the poop test because that’s all the doctor said to do. She said, we’ll do one step at a time. So if anything turned up with the stool test, they would do a colonoscopy.* (Kin #25, Precontemplation –GP sent letter)

Lastly, many FDRs reported that their HCPs simply did not recommend colonoscopy to them or did not follow-up with their request to undergo one. For example, one FDR mentioned perceiving that her GP did not recommend colonoscopy based on her age (> 75):

*When I told my GP, I don’t think she insisted that I have a colonoscopy. And certainly, no other doctor has ever told me I should have one or should have had one three years ago.* (Kin #19, Precontemplation –GP not sent letter)

#### Practical concerns and inconvenience

FDRs brought up several practical concerns surrounding colonoscopy such as scheduling, forgetfulness, taking time off work, bad weather, arranging a ride, and other competing responsibilities (i.e., being caretaker for a parent and job responsibilities). For example:

*Oh, there’s no benefits at all to not having it. Other than having to work that into my schedule and having to deal with either having it here and getting okay with that idea with my work colleagues or going out of town.* (Kin #14, Precontemplation, -GP sent letter)

*Scheduling would be number one and then I’m forgetful, number two (*Kin #2, Contemplation, -GP sent letter)

#### Lack of information

Many FDRs reported a lack of information that acted as a barrier to colonoscopy in the following realms: understanding risk; misinformation about CRC; on colonoscopy in general; and in regard to preferences for information.

FDRs believed their risk for CRC to be low or negligible, and sometimes attributed familial risk to their spouse’s side of the family when applicable. One FDR reported perceiving her risk to be low based on older age and the fact that she believed risk to be on her spouse’s side:

*No, because, perhaps wrongly so, but I don’t feel I’m at risk… I’m not going to worry about this because I don’t think I’m at risk that much.* (Kin #19, Precontemplation –GP not sent letter)

Although most FDRs discussed being aware of their increased risk, many were not sure what this meant and desired more information on their specific risk profile.

*I mean, what are the odds, 1 out of 8 kids got colon cancer. What are the odds of the rest of us 8 getting it?* (Kin #25, Precontemplation –GP sent letter)

Misinformation (or lack of accurate information) about CRC included the belief that FOBT was a sufficient method of screening, and the belief that CRC would present with symptoms. For example, some FDRs discussed their belief that because they were asymptomatic, their risk for CRC was low and therefore they did not need to undergo colonoscopy. Although most FDRs discussed understanding that CRC can present without symptoms and acknowledged the risk of assuming otherwise, the presence of bowel symptoms still played a role in the decision not to undergo colonoscopy, for example:

*Well if I developed symptoms that were concerning then I’d be more likely to do it although then it wouldn’t be a screening probably*. (Kin #14, Precontemplation –GP sent letter)

FDRs also described a lack of information about colonoscopy in general and expressed a desire for procedure-specific information such as how to be referred for one and how often someone should be screened, despite this information being addressed in the letter that all participants received as part of this study. FDRs also discussed being unaware of where to find reputable information about colonoscopy, and uncertainty regarding the difference between FOBT and colonoscopy.

Some FDRs reported a preference for ignorance regarding CRC and talked about the bad news that colonoscopy could bring. Many FDRs also described the distress that colonoscopy can create, in line with the idea that “ignorance is bliss”:

*If you don’t know, then you’re fine. If you do know, then you have to start worrying about or thinking about what you need to do.* (Kin #9, Precontemplation –GP not sent letter)

*Not performing a colonoscopy is like acting like an ostrich, putting my head in the sand and avoiding what is.* (Kin #2, Contemplation –GP sent letter)

## Discussion

Both probands and FDRs identified several important barriers to disclosure and colonoscopy adherence. Consistent with existing literature, common barriers discussed by probands included lack of information, negative expectations about familial reaction, assumptions that FDRs were already aware of risk or already being screened, cohort effects, and dysfunctional family dynamics. Also consistent with other literature, common barriers discussed by FDRs included negative attitudes about colonoscopy, lack of motivation, HCP-related factors, practical concerns and inconvenience, and lack of information. Novel to this study was the insight provided into the decision-making process about disclosure and screening by probands and FDRs through the lens of the TTM.

### Proband disclosure

Little research has focused specifically on the decision making process probands undergo when disclosing to FDRs that they require colonoscopy. The current study found that although the majority of probands disclosed to most of their FDRs, many probands had not told at least one FDR, and 25% had told no FDRs at all.

Consistent with the barriers reported in this study, Katz et al. found that patients who had undergone genetic testing did not discuss their results with kin for many reasons including not having contact with the relative, assuming other kin had told that relative, and believing the relative was too old for the information to be useful [25]. Also consistent with our findings, a review examining communication following genetic testing for hereditary cancers showed that probands were more likely to disclose information if they had close relationships with kin, had a sense of responsibility towards kin, felt the information was relevant to the kin and would be received well, and/or their HCPs had encouraged them to do so [14]. We found that cultural barriers impacted the decision to disclose to FDRs for some probands who discussed stigma surrounding the topic of cancer in general, and also discussed differences in health screening practices across cultures.

This study provides novel insights into how motivated probands are to engage in disclosure. For those in the Precontemplation stage, who had no intention of disclosing to their FDRs, reasons for nondisclosure included negative expectations about familial reactions, estrangement and dysfunctional family dynamics, lack of correct information about CRC risk, and cultural barriers to family communication. Probands in Precontemplation who reported estrangement and dysfunctional family dynamics as central barriers often endorsed the belief that either other family members had informed the individual(s) who they were unwilling to contact, or that the estranged family member(s) may have already taken steps to be screened, making contact with them unnecessary.

Our research revealed a deeper and more nuanced understanding of the barriers to disclosure for probands in the Precontemplation stage, and provided insight into how family dynamics, cultural barriers, and probands’ expectations of FDRs response can hinder communication about the need for colonoscopy.

### FDR adherence to colonoscopy

Although all FDRs received a letter about their increased risk for CRC and need for colonoscopy, the majority were in the Precontemplation Stage of Change and discussed no intention of undergoing colonoscopy. This finding was especially striking because three of the five FDRs in Precontemplation also had letters sent to their GPs about their increased risk for CRC and need for colonoscopy as part of the study.

FDRs in Precontemplation had negative attitudes about colonoscopy preparation and the procedure itself, received erroneous information from their GPs about their risk or need for colonoscopy, had practical concerns, and reported a lack of information. For the two FDRs in the Contemplation Stage of Change, scheduling concerns and lack of understanding about their risk were recurrent themes.

Our findings are similar to those reported by Rawl et al. who found that 64% of FDRs of probands with CRC were in the Precontemplation stage regarding the decision to undergo colonoscopy [8]. FDRs in their study reported barriers such as not having a HCP recommend colonoscopy, lack of understanding about colonoscopy, and embarrassment. These barriers, as well as fear of CRC, inconvenience, perceived invulnerability, and cost have been identified in other research on FDRs of probands with CRC [11]. Of these variables, cost and lack of GP recommendation have been identified as two of the most important barriers to colonoscopy [7, 26]. Interestingly, when these barriers are irrelevant, as they were in the present study’s Canadian sample who have access to free healthcare and whose GPs were informed about their increased risk for CRC, FDRs still report significant challenges to undergoing colonoscopy.

### Clinical implications

The barriers identified in this study would be difficult for both patients and HCPs to overcome during a short office visit. Several studies have shown that using motivational interviewing (MI), which uses the TTM to tailor psychological interventions based on the patient’s stage of change to increase motivation for behavior change, can be helpful for increasing colonoscopy uptake in FDRs of individuals with CRC [27–29]. For example, a randomized clinical trial of FDRs of probands under age 60, which delivered telephone MI based on the participants’ Stage of Change, showed a 32% increase in colonoscopy adherence post-intervention [27]. Similar findings have been reported by Kinney and colleagues [28], while another trial conducted in Iran showed an increase in colonoscopy uptake of 83.5% [29]. An important benefit of a MI approach is that it can be delivered in just one or two sessions and data show that it is cost effective [28]. It can also be delivered by any HCP, if they have been trained in this approach.

While we are unaware of existing research on the impact of MI on disclosure to FDRs among probands, given its success in increasing colonoscopy adherence, future research should explore the impact of MI on disclosure. Other tools have also been suggested to improve disclosure. For example, in Denmark, unsolicited letters with information about hereditary CRC were sent to FDRs of people diagnosed with LS [30]. These letters were well-received by the majority of kin, although 40% of participants stated they would have desired the information from a close relative instead. However, most healthcare laws prevent the use of unsolicited letters to kin and presently the burden of disclosure falls squarely on probands. Chivers Seymour et al. proposed a script for genetic counselors to use with probands to encourage family communication following genetic testing and although no efficacy data are available, this tool could be adapted and tested in future research with probands who need to communicate with FDRs about CRC risk [14].

### Limitations

A number of study limitations should be noted. First, the method we used to recruit probands was not a good proxy for actual nondisclosure. Only probands who did not grant consent to the study team to contact their FDRs were approached for participation. While this strategy did provide access to several probands who were in the Precontemplation Stage of Change for disclosure, many others were already in the Action or Maintenance stage with most or all of their FDRs. The response rate was also very low however, it is challenging to recruit people who are not engaging in health behaviours (i.e., disclosure of CRC risk; colonoscopy) for a study focused on why they are not taking action. Given that participants were willing to be interviewed about this difficult topic, our data may actually underestimate the degree of reluctance to engage in these health behaviors. Further, our sample was small, relatively young, and located within the Canadian universal healthcare system, and therefore findings from this study may not generalize to other populations.

## Conclusions

Despite these limitations, this study provides a novel perspective on both probands who received standardized information about the necessity of discussing CRC risk and need for colonoscopy with their FDRs, and on FDRs who also received standardized information about the need for colonoscopy. It is clear that information is not enough for both groups, as a number of important barriers were identified for the most unmotivated probands and FDRs. HCPs working with these populations should consider using empirically-supported approaches, such as MI, to reach probands and FDRs who struggle with these healthcare decisions.

## Data Availability

The datasets used and or analyzed during the current study are not publicly available due to privacy for participants in this small qualitative dataset, but are available from the corresponding author on reasonable request.

## Abbreviations

C4: Canadian Colorectal Cancer Consortium
CRC: Colorectal Cancer
FAS: Family Address Sheet
FDR: First degree relative(s)
FOBT: Fecal occult blood test
GP: General practitioner
HCP: Health care provider
LS: Lynch syndrome
MI: Motivational interviewing
TTM: Transtheoretical model
